# Numerical Modeling of an Organic Electrochemical Transistor

**DOI:** 10.3390/bios8040103

**Published:** 2018-10-31

**Authors:** Anna Shirinskaya, Gilles Horowitz, Jonathan Rivnay, George G. Malliaras, Yvan Bonnassieux

**Affiliations:** 1 Laboratory of Physics of Interfaces and Thin Films (LPICM), Ecole Polytechnique, Route de Saclay, 91128 Palaiseau CEDEX, France; gilles.horowitz@polytechnique.edu (G.H.); yvan.bonnassieux@polytechnique.edu (Y.B.); 2 Department of Bioelectronics, Ecole Nationale Superieure des Mines CMP-EMSE MOC, 13541 Gardanne, France; jrivnay@northwestern.edu (J.R.); gm603@cam.ac.uk (G.G.M.); 3 Department of Biomedical Engineering, Northwestern University, 2145 Sheridan Road, Evanston, IL 60208-3109, USA; 4 Electrical Engineering Division, Department of Engineering, University of Cambridge, 9 JJ Thomson Ave, Cambridge CB3 0FA, UK

**Keywords:** organic electrochemical transistor, biosensor, model, de-doping, moving front

## Abstract

We develop a numerical model for the current-voltage characteristics of organic electrochemical transistors (OECTs) based on steady-state Poisson’s, Nernst’s and Nernst–Planck’s equations. The model starts with the doping–dedoping process depicted as a moving front, when the process at the electrolyte–polymer interface and gradually moves across the film. When the polymer reaches its final state, the electrical potential and charge density profiles largely depend on the way the cations behave during the process. One case is when cations are trapped at the polymer site where dedoping occurs. In this case, the moving front stops at a point that depends on the applied voltage; the higher the voltage, the closer the stopping point to the source electrode. Alternatively, when the cations are assumed to move freely in the polymer, the moving front eventually reaches the source electrode in all cases. In this second case, cations tend to accumulate near the source electrode, and most of the polymer is uniformly doped. The variation of the conductivity of the polymer film is then calculated by integrating the density of holes all over the film. Output and transfer curves of the OECT are obtained by integrating the gate voltage-dependent conductivity from source to drain.

## 1. Introduction

First reported by Mark S. Wrighton in 1984 [1], the organic electrochemical transistor (OECT) has seen redevelopment twenty years later, with the advent of poly(styrene sulfonate) doped poly(3,4-ethylenedioxythiophene) (PEDOT:PSS) [2]. Unlike most conducting polymers, PEDOT:PSS is conducting in its pristine form, and may be turned to insulating when doped with a cation. One reason for the success of this compound in OECTs is that it is commercially available under different forms, including thin film deposited on plastic foils. PEDOT:PSS based OECTs are currently actively studied for various types of applications, such as efficient signal amplifiers [3], electrochemical logic circuits [4], in vivo neural recording devices [5] and chemical sensors [6]. Besides this wide range of applications, the use of OECTs as biosensors has emerge as particularly fruitful. Functionalized OECTs have already proven to be used for glucose [7,8,9], lactate [10], liposome [11], dopamine [12], DNA [13], bacteria [14] detection and as well as for ultrasensitive detection of proteins, such as Immunoglobulin G (IgG) [15]. The latest review paper by J. Rivnay [16] gives a very good overview of the current and future use of OECTs.

An OECT consists of a conducting polymer layer equipped with two electrodes, source and drain, in contact with an electrolyte. A third electrode, the gate, is immersed in the same electrolyte. The conductivity of the polymer film can be monitored by applying a voltage between the source and the gate, through a process of reversible doping and dedoping. In the case of PEDOT:PSS, the film is conducting when no bias is applied, and become insulating when applying a source-gate bias, making the device a normally on transistor. The high conductivity of doped PEDOT:PSS also allows for its use to make source, drain and gate electrodes, thus offering a high versatility for the fabrication of the device [4].

OECT fundamentally differs from field-effect transistors in that the switching mechanism does not initiate through the formation of a thin conducting channel at the semiconductor–insulator interface. Instead, the conductivity of the whole polymer film is changed during the doping–dedoping process. There are several aspects that make modeling of the doping–dedoping process difficult. Basically, conducting polymers are degenerately doped semiconductors, with a density of dopants ranging between 10^18^ and 10^21^ cm^−3^. However, they also behave as ionic conductors. Moreover, the doping–dedoping mechanism involves an electrochemical reaction that occurs in the bulk of the polymer layer [17,18]. Such a mechanism is difficult to describe with conventional electrochemical models because, in principle, electrochemical reactions take place at the interface between an electrode and an electrolytic solution.

A fruitful concept for resolving this difficulty is that of the “moving front”, first introduced by Tesuka and Aoki in 1989 [19], when a doped domain propagates through the polymer film, starting from the polymer-electrolyte interface. Since then, several theoretical studies have been carried out to account for the moving front model [20,21,22]. More recently, Stavrinidou and coworkers conducted experiments to show the applicability of the model to the mechanism of doping–dedoping in PEDOT:PSS [23]. For this, they fabricated a planar junction by depositing the conducting polymer on a parylene substrate. Because PEDOT:PSS changes its color from almost transparent to deep blue upon dedoping, the process could be optically followed. What they found is that dedoping starts at the electrolyte side of the junction, and that a dedoping front gradually moves from the electrolyte to the base electrode.

However, in spite of its primary usefulness in elucidating the doping–dedoping process in conducting polymer, the moving front model is less interesting when it comes to model the OECT because it mainly restricts to the transient stage of the process, while modeling the OECT requires knowledge of the state of the polymer at steady-state. In particular, the available models fail to give a response to the basic question: Where does the moving front stop when steady state is reached if it ever stops somewhere before the base electrode?

Modelling of OECT is a very important instrument that allows us not only to understand the device working principle, but also to model its performance. There exist several OECT models with different degree of complexity, each of them looking at OECT from its own perspective, but a lot of them represent an OECT from the point of view of physical generalization. First a purely theoretical model was developed by Prigodin et al. [24] in 2005, which assumes that the decrease of current is due to a decrease of hole mobility, caused by the penetration of ions inside the conductive polymer. Being purely theoretical, this model does not allow data fitting. In contrast, the second model by Robinson et al. [25] proposed to look at an OECT from an electrochemical and electrostatic point of view, and linked the drop in conductivity to the applied potential induced dedoping process leading to cations penetration and the following hole extraction. This model is numerical and also does not allow the straightforward data fitting and parameters extraction.

Many attempts to rationalize the operation of OECTs also start from a dynamical model based on a time-dependent Nernst–Planck equation approach. Among them, we can cite the works by Volkov et al. [26] and Tybrandt et al. [27]. Together with the moving front approach, these models are useful to elucidate the transient behavior of the OECT, but lack pertinence when it comes to model the steady state operation of the device. In parallel to these time-dependent approaches, Malliaras’s group focused on purely electrostatic models, which bring useful information as far as the geometry of the device is concern, but fail to correctly account for its current–voltage characteristics because they do not include a correct description of the doping–dedoping mechanism [28,29].

In this paper, we develop a model to account for the specificities of the OECT. In particular, we restrict our calculation to the steady state reached after applying a source-gate voltage to the device. In a first stage, numerical calculations are performed with the COMSOL Multiphysics package to resolve the basic equations of the model. These calculations are made on a 1D device, only including the source and gate electrodes. This leads us to three possible descriptions of the modulation of the conductivity of the polymer by the applied source-gate voltage. Experimental data, using a modified setup of that developed by Stavrinidou [23], helped us in selecting the correct description. In the second stage, the graded channel approximation is used to calculate the current–voltage curve of the transistor.

## 2. Materials and Methods

Photolithography in the clean room was chosen as a method to fabricate OECTs as well as steady state potential measurement devices, this method was described precisely at [3]. Electronically conductive channel was fabricated of PEDOT:PSS water dispersion (PH1000 from from the Heraeus Clevios). (3-Glycidyloxypropyl)trimethoxysilane (0.1 wt %) was added to the dispersion for the film stabilization and adhesion to the substrate. Drain electrode of OECT, source electrodes and potential probes were made of Au and deposited by evaporation; finally contacts were insulated by Parylene-C. Ag/AgCl electrode was used as a gate electrode of OECTs. 100 mM NaCl in DI water solution was used as electrolyte solution. Electrical measurements and device characterization was made according to the protocols described previously [3]. 

## 3. Results and Discussion

### 3.1. Model Description

#### 3.1.1. Physical Description of the Doping–Dedoping Process Occurred in the Channel of an OECT

**Figure 1:**
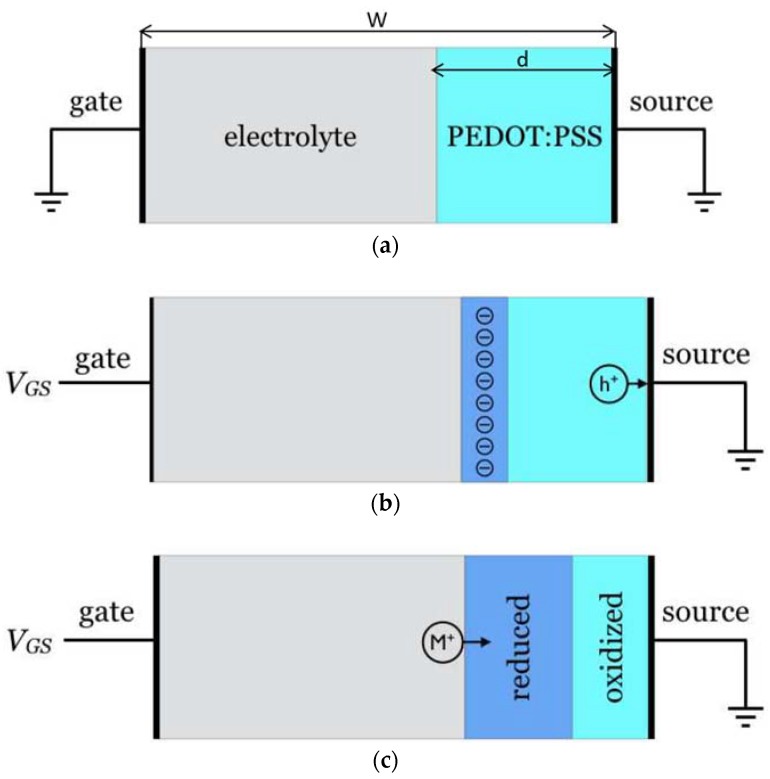
Structure of the OECT (**a**) at equilibrium (no voltage applied); (**b**) upon application of a gate-source voltage *V_GS_*, holes h^+^ leave the polymer. Because of the high concentration of holes in the oxidized polymer, this leads to the formation of a thin space charge layer at the electrolyte–polymer interface; then (**c**) driven by the electric field in the space charge layer, M^+^ cations penetrate from the electrolyte, thus warranting electrical neutrality in the polymer. As a result, the profile of the reduced layer is profoundly reshaped, and could ultimately occupy the whole thickness of the polymer.

In contrast with field-effect transistors, where the current modulation originates from the change in the interfacial charge density, the modulation of the drain current in OECTs has its origin in a modulation of the conductivity of the conducting polymer layer. In the first step of the process, we consider the one-dimensional structure depicted in Figure 1, with a fully oxidized PEDOT:PSS layer inserted between a source electrode and an electrolyte. The structure is completed by a gate electrode in contact with the electrolyte.

The doping–dedoping process of the polymer is controlled by the redox reaction given by Equation (1), where *M*^+^ is the cation present in the electrolyte.

(1) $$\left(PEDOT^{+}:PSS^{-}\right)+M^{+}+e^{-}↔PEDOT^{0}+\left(M^{+}:PSS^{-}\right),$$

Evidence for electron exchange taking place in reaction (1) is brought by the presence of a transient current through the gate electrode. Importantly, the charge associated with the oxidized form of PEDOT is mobile; we shall call this charge a hole.

When a bias is applied to the gate, holes leave the polymer and initiate a redox reaction at the interface between the gate and the electrolyte. For the sake of simplicity, we assume that the volume of the electrolyte is much larger than that of the polymer, so that the limiting redox process is that in the PEDOT:PSS layer. We also assume that there is little voltage drop at the gate–electrolyte interface, so that at steady state, the potential at the electrolyte–polymer interface is equal to that at the gate. Keeping the source grounded, the potential in the polymer film gradually increases from zero to *V_GS_*, where *V_GS_* is the gate-source bias. When *V_GS_* is above a given threshold *V_T_*, reduction of PEDOT occurs beyond a given point, and an insulating (reduced) polymer layer grows at the polymer-electrolyte interface. For the sake of electrical neutrality, cations from the electrolyte penetrate in the polymer. At steady state, the situation is that depicted in Figure 1c: the polymer is divided into a conducting layer near the source and an insulating (reduced) layer near the electrolyte.

#### 3.1.2. Basic Equations

Since we are only interested in a steady state description of the device, the numerical simulation only uses static equations: Nernst, Poisson and the electrical neutrality. Equations like Nernst–Planck, that describe the movement of ions, will not be involved. Also, the transient gate current that accompany reaction (1) is outside the scope of our analysis.

The electrical potential *v* in the polymer is connected to the respective density of oxidized and reduced PEDOT through Nernst’s equation:

(2) $$v=v^{0}+\frac{kT}{q}ln\frac{\left[ox\right]}{\left[red\right]},$$

where *v*^0^ is the redox potential, [*ox*] and [*red*] the density of oxidized and reduced PEDOT, respectively, *k* is Boltzmann’s constant, *T* the temperature and *q* the elemental charge. Calling *c_p_* the density of PEDOT^+^ (which is also the density of holes) and *c*_0_ the total density of PEDOT (which is also the density of PSS^−^), the density of reduced PEDOT is *c*_0_ − *c_p_*, and (2) can be rewritten as:

(3) $$c_{p}=\frac{c_{0}}{1\;+\;exp\left[-\frac{q}{kT}\left(v\;-\;v^{0}\right)\right]},$$

Here, *v* and *v*^0^ correspond to potentials of the working electrode of a conventional three-electrode electrochemical cell; they are measured against a reference electrode. However, in a transistor, the source is usually connected to ground, the varying potential is that at the gate, and there is no reference electrode. Accordingly, we rewrite (3) by reverting the sign of the potential:

(4) $$c_{p}=\frac{c_{0}}{1\;+\;exp\left[\frac{q}{kT}\left(V\;-\;V_{T}\right)\right]},$$

Here, *V* is measured as a function of the grounded source electrode. Because a conducting polymer can be viewed as a degenerate semiconductor, the potential at the source is close to the HOMO edge. The threshold voltage *V_T_* corresponds to the difference between the redox potential and the work function of PEDOT:PSS. Because the polymer is highly doped, the Fermi level is close to the HOMO edge and *V_T_* can be expected to be small.

The total electric charge is made of three components: (1) PSS which induces a fixed charge of density *c*_0_, (2) mobile holes with the concentration given by Equations (4) and (3) mobile cations *M^+^* with a concentration *c_M_* that warrant global electrical neutrality in the polymer layer. The movement of ions in the polymer can be mathematically represented by using Nernst–Planck equation (also known as drift–diffusion equation). At steady-state, this equation writes:

(5) $$qc_{M}\mu F\;-\;qD\frac{dc_{M}}{dx}=\;0,$$

Here, *F* is the electric field, *μ* the mobility of the cation and *D* their diffusion coefficient, which is connected to *μ* through Einstein’s relation *D*/*μ* = *kT*/*q*.

The last constraint is given by Poisson’s equation that relates the potential to the charge density *ρ = c_P_ +c_M_ − c*_0_:

(6) $$\frac{d^{2}V}{dx^{2}}\;=\;-\frac{\rho \left(x\right)}{\varepsilon },$$

As will be shown in the following, the results of the calculation strongly depends on the way the cations move in the polymer before reaching steady state. Based on what fund in the literature, we developed two different models.

In the first model, the reduction of PEDOT occurs when a cation reaches an oxidized site, and the ion remains trapped at this site [21]. In other word, each time an oxidized PEDOT^+^ is reduced to PEDOT^0^, it is replaced by a cation that remains there, thus maintaining local electrical neutrality:

(7) ρ(x) = 0,

Hence, we will call this first model “local neutrality” model.

In the second model, we assume that cations remain free to move all over the polymer layer after inclusion. Of course, electrical neutrality must be globally maintained all over the polymer:

(8) $$\int_{0}^{d}\rho \left(x\right)dx\;=\;0,$$

where *d* is the thickness of the polymer film. We will call this second model ‘global neutrality’ model.

### 3.2. Model Implementation and Experimental Results

#### 3.2.1. Steady-State Potential and Hole Density Profile

All our calculations were performed using the COMSOL Multiphysics software, equipped with the electrochemistry package. The calculations were implemented on the 1D structure shown in Figure 1a,b. The distance between two electrodes is *W*. The grounded source electrode is located at right hand side, so *V*(*W*) = 0 V. Gate electrode is place at the left hand side, so *V*(0) = *V_GS_*. In between two electrodes there are an ionically conductive layer that contains Cl^−^ and Na^+^ ions and an electronically conductive PEDOT:PSS layer, with thickness d, into which Na^+^ penetrate when dedoping occurs. The modeled device consists of two layers:

1. An ionic layer with 100 mM of NaCl solution. The thickness of the layer is *W* − *d* = 900 nm.
2. An electronically conductive PEDOT:PSS layer. The initial density of PEDOT^+^ (mobile holes) equals that of PSS^−^ (immobile anions) was set to 10^18^ cm^−3^ which is probably lower than the actual density in real devices. This choice of initial hole density was made on purpose to make clearer the correlation between hole density inside conductive polymer and potential applied. The thickness of the conductive polymer layer is *d* = 100 nm. This thickness was chosen in reference to actual OECT biosensors.

**Figure 2:**
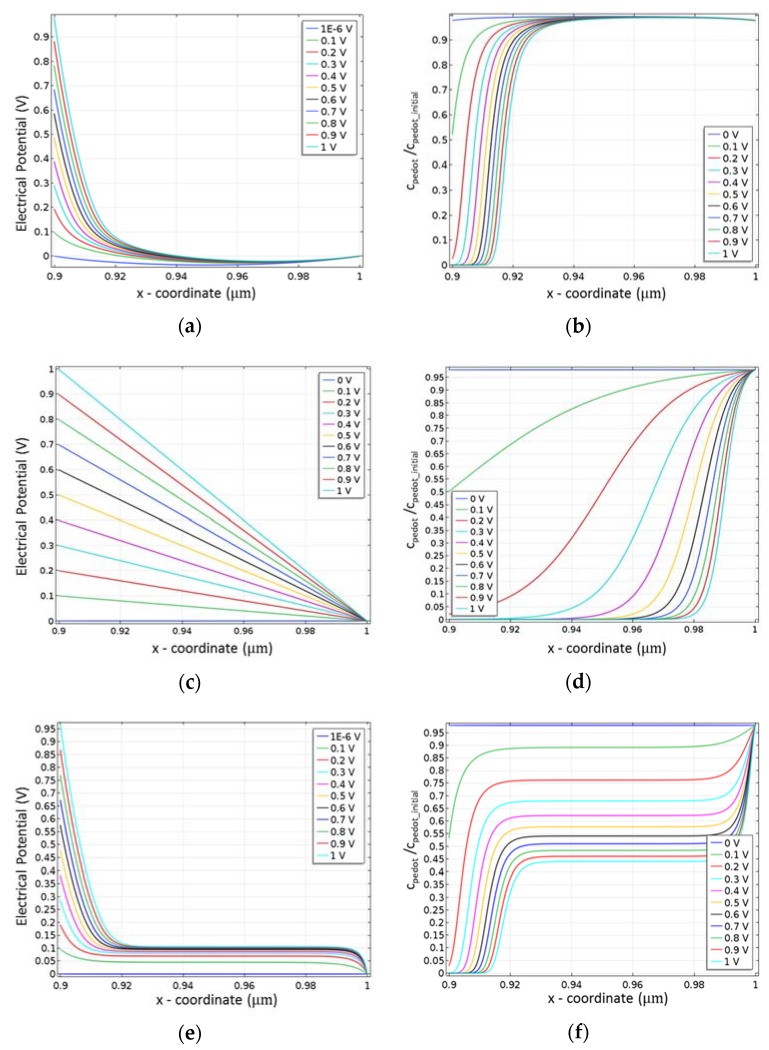
Calculated steady state data of the PEDOT:PSS layer at various applied gate-source voltages (from 0 V to 1 V, with 0.1 V step): (**a**,**c**,**e**) potential along the conductive polymer layer; (**b**,**d**,**f**) hole concentration profile. The potential and hole concentration was numerically evaluated in three cases: (**a**,**b**) no penetration of the cations; (**c**,**d**) local electrical neutrality; (**e**,**f**) global electrical neutrality.

The potential and hole concentration were numerically evaluated in three cases: (1) no penetration of the cations; (2) local electrical neutrality and (3) global electrical neutrality (Figure 2).

In view of the numerical data, it is possible to draw the following observations for each case:

1. In the case of no ion penetration inside the conductive polymer (Figure 2a,b) the main potential drop and reduction of PEDOT^+^ concentration occurs at the interface with the electrolyte. The reduced part of PEDOT:PSS layer widens when the applied potential increases. Nevertheless, even at a relatively high applied potential (1 V), only a small part of the channel is reduced. Due to such a small influence of the applied potential on PEDOT^+^ concentration this model is not appropriate to describe both the moving front experiment and OECT behavior.
2. In the case of Local electro-neutrality, the drop of the potential is linear along the thickness of the channel, which perfectly fits the expected zero electric field gradient profile (Figure 2c). Figure 2d represents the concentration profile of PEDOT^+^. As expected, this concentration decreases with the applied source-gate voltage, and the shape of the decrease highly resembles the moving front experiment profile [30]. However, it must be pointed out that we are here dealing with a steady state model, so the front does not move with time, but instead with the applied gate-source voltage. To the best of our knowledge, no experimental data in the literature would confirm or reject such a behavior.
3. In the case of Global electro-neutrality (Figure 2e,f) the main potential drop occurs at the very interface between the electrolyte and conductive polymer. An additional potential drop occurs near an interface between the conductive polymer and Source electrode. In between, that is, in most of the conductive layer, the potential profile is flat and saturates at around 0.1 V for all applied Gate-Source voltages from 0 V to 1 V.

#### 3.2.2. Experimental Check

**Figure 3:**
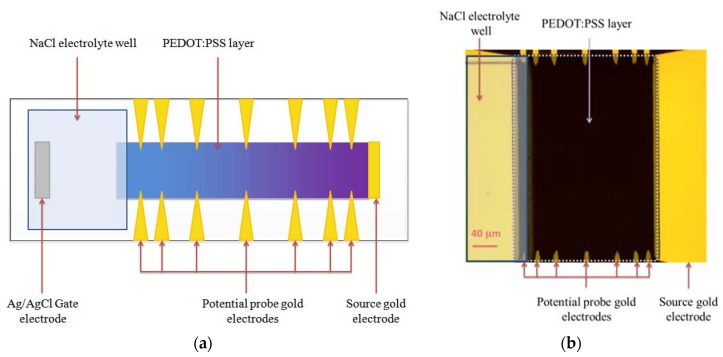
Experimental set up for steady state potential measurement of PEDOT:PSS channel under applied gate-source potential: (**a**) Simplified schematic representation; (**b**) Real image.

To determine which of the two numerical models (Local or Global electro-neutrality) is correct, the experimental set up shown in Figure 3 was carried out.

The device consisted of Ag/AgCl gate electrode immersed in a well filled with the NaCl electrolyte with concentration 100 mM; the PEDOT:PSS layer was connected to the electrolyte layer at one side and to the gold Source electrode at the other side. Fourteen probe electrodes were connected to the PEDOT:PSS layer at both sides of the channel to measure the electrical potential at various distances from the source electrode. Thus, the device allows measuring the potential along the channel to compare the experimentally measured potential profile with that which was numerically calculated. The device has the following geometrical characteristics: the thickness of PEDOT:PSS layer is equal to 100 nm, the width—200 µm and the length—200 µm.

**Figure 4:**
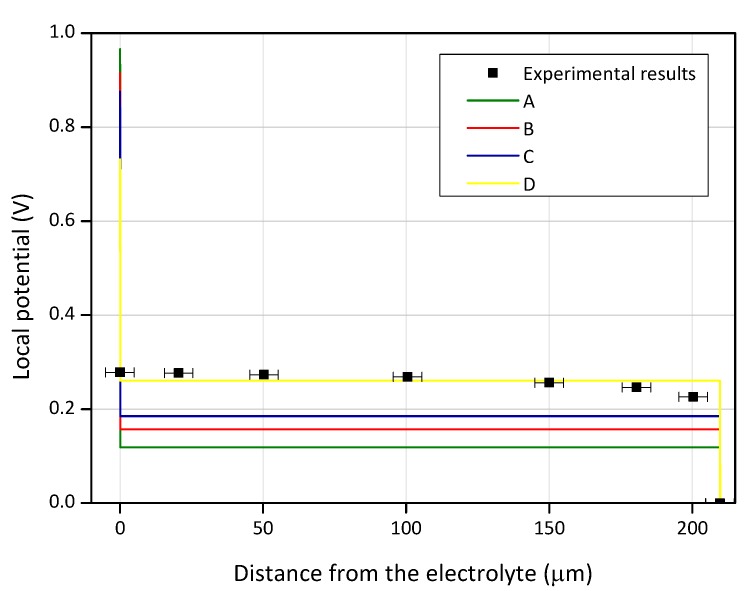
Experimentally measured (marked line) and numerically calculated (straight line) potential profile along the PEDOT:PSS channel. The calculated profiles were computed with various initial hole concentrations: **A**—10^18^ cm^−3^; **B**—5·10^18^ cm^−3^; **C**—10^19^ cm^−3^; **D**—5·10^19^ cm^−3^.

The experimental results are summarized in Figure 4. It is clear that the measured profile shows high similarity with that modelled with the ‘global neutrality’ assumption. This proves that the “global neutrality” model is correct and reflects well the real situation in an OECT. The difference between the calculated and measure values in the channel could be explained by the difference between the actual hole density and that used as an input for the numerical simulation. When correcting this parameter, it is possible to get an almost perfect match between the numerically calculated and experimentally measured potential profiles.

Figure 4 clearly shows that potential profile in the channel depends not only on the applied voltage, but also on the initial hole concentration. According to the fit between experimental and calculated data, the initial hole concentration is close to 5 × 10^19^ cm^−3^.

An additional argument in favor of the local neutrality model is brought by a recent work by Modarresi et al. [31], in which no sign of ion trapping was observed.

#### 3.2.3. Output and Transfer Curves of the OECT

**Figure 5:**
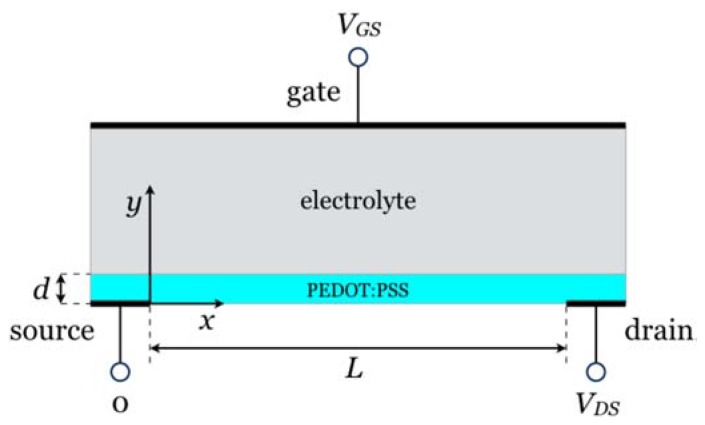
General view of an OECT. *x* and *y* axes correspond to the directions along and across the polymer film, respectively.

As depicted in Figure 5, the transistor is a three-terminal, two-dimensional device, with two independent applied voltages, one between source and gate (*V_GS_*) and the other one between source and drain (*V_DS_*). 

To calculate the drain current, we make use of the well-documented gradual channel approximation [32], which is based on the fact that, because there is a current flowing between source and drain, the potential at the free interface of the polymer layer (*y* = 0) gradually varies from its value at source to that at drain. As a consequence, although the polymer is not in direct contact with a metal (at variance with the 1D structure), the potential at *y* = 0 can be viewed as imposed by externally applied voltage sources (like in the case of the 1D structure) and slowly varying between source and drain. The gradual channel approximation also states that, because the distance L from source to drain (also known as channel length) is much larger that the thickness d of the polymer layer, the electric field across the layer (generated by *V_GS_*) is much larger than that along it (generated by *V_DS_*). Accordingly, the conductivity can be calculated as in the case of the one dimensional structure analyzed in the previous section.

We first calculate the conductivity σ of the polymer layer with no source-drain voltage applied. This is given by:

(9) $$\sigma \;=\;qc_{p}\mu ,$$

Here, *q* is the elemental charge and *μ* the hole mobility. The variation of the conductivity with the source-gate voltage may have two origins: the variation of the integrated density of holes, and a variation of the mobility, which is often reported to increase with hole density. However, we note that in our two-layer model, the variation of the hole density remains close to its maximum value in the conducting region, so one can expect the effect of a hole density-dependent mobility will have a limited impact on the conductivity.

Assuming constant mobility, the relative conductivity writes:

(10) $$\frac{\sigma \left(V_{GS}\right)\;}{\sigma_{max}}\;=\;\int_{0}^{d}\frac{c_{p}\left(V_{GS},\;x\right)}{c_{0}}dx,$$

Here, *σ_max_* = *qc*_0_*μ* is the conductivity of the fully undoped polymer.

**Figure 6:**
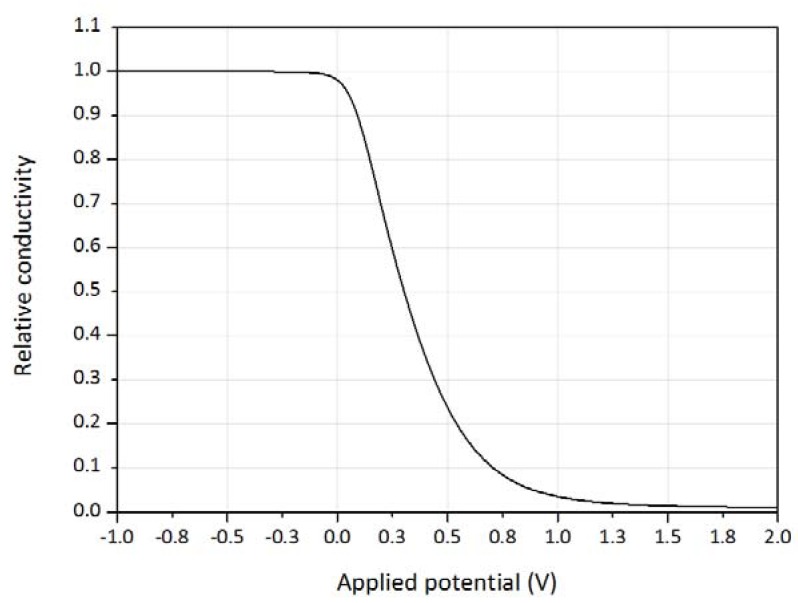
Calculated gate-source voltage dependent relative conductivity of PEDOT:PSS, obtained by integrating the data in Figure 2f.

The source-gate voltage dependent conductivity corresponding to the data in Figure 2f is shown in Figure 6.

We now apply a voltage between source and drain. To calculate the drain current, we start with Ohm’s law:

(11) $$j_{D}\;=\;\sigma F,$$

where *j_D_* is the drain current density and F the electric field along the channel. Writing *F* = *−dV*/*dx*, (11) becomes:

(12) $$j_{D}dx\;=\;-\sigma \left(V\right)dV,$$

We define the *x* axis as pointing from source to drain, with an origin at the source. The conductivity at a point *x* from the source depends on the potential at the polymer-electrolyte interface at this point. This potential is modulated by the drain voltage *V_DS_* from *V_GS_* at source (*x* = 0) to *V_GS_* − *V_DS_* at drain (*x* = *L*), where *L* is the channel length.

Noting that the current is conservative, (12) can be integrated from source (*x* = 0, *V* = *V_GS_*) to drain (*x* = *L*, *V* = *V_GS_* − *V_DS_*) as follows:

(13) $$j_{D}\int_{0}^{L}dx\;=\;j_{D}L\;=\;-\int_{V_{GS}}^{V_{GS}-V_{DS}}\sigma \left(V\right)dV,$$

The drain current *I_D_* is obtained by multiplying the drain current density by the cross section of the polymer layer, that is, the width of the channel *W* times the thickness *d* of the film. It comes:

(14) $$I_{D}\;=\;j_{D}Wd\;=\;-WdL\;\int_{V_{GS}}^{V_{GS}-V_{DS}}\sigma \left(V\right)dV,$$

Note that for a hole conducting polymer like PEDOT:PSS, *V_GS_* > 0, while *V_DS_* < 0. Also, the conductivity deceases when *V_GS_* increases.

**Figure 7:**
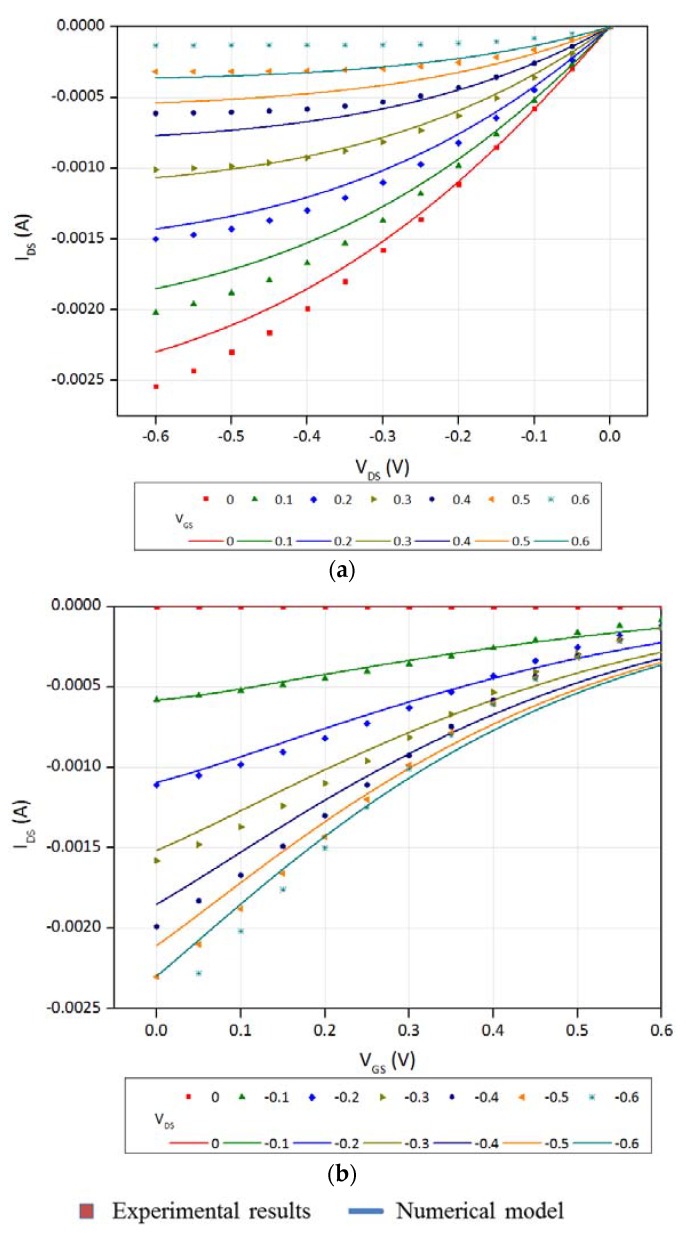
Calculated output (**a**) and transfer (**b**) curves of an OECT. The parameters used for the calculation are gathered in Table 1.

Numerically calculated current–voltage curves for one device were compared with experimental data to check the validity of the 1D model and the possibility to use it for the real device parameter calculations. Calculated and measured output (*I_D_* − *V_DS_*) and transfer (*I_D_* − *V_GS_*) curves are drawn in Figure 7. The values of the parameters are gathered in Table 1.

Figure 7 shows a fair agreement between our model and the experimental data. The best agreement is found in the transfer curves at low source-drain voltage (Figure 7b). In spite of the deviations that appear at higher voltages and in the output curves, we can reasonably state that the global neutrality model fairly describe the OECT operating mode. The origin of the deviations could be found in the presence of several additional effects that were not taken into account in our model, e.g., movement of ions under the effect of the drain-source voltage.

## 4. Conclusions

Theoretically based modelling is a very powerful tool to describe the working principle of an OECT and quantitatively characterize the device. The usage of the models is not only the matter of a global understanding, but is also a prominent step towards an optimal and efficient device creation.

In this work, three different models were proposed: “no-ions penetration” model; “local neutrality” model and “global neutrality” model. A clear proof of the “global neutrality” model validity was shown by matching experimentally obtained local conductive channel profiles with those calculated numerically. According to this model, the ions penetrating from the electrolyte inside the conductive polymer layer are not locally trapped; rather, they are moving freely inside the polymer layer, maintaining only global neutrality over the whole volume or the polymer. It was demonstrated that using the global neutrality modeled channel conductivity allows to obtain drain current profiled under different applied drain-source and gate-source potentials. The calculated profiles show reasonable agreement with the experimentally measured profiles for real OECT devices, which proves that the “global neutrality” model could be used for a device characterization and description as well as for its behavior prediction. Remaining discrepancies could be attributed to features not taken into account in the model, such as concentration-dependent hole mobility.

## Figures and Tables

**Table 1:** Parameters used for the calculation of the output and transfer curves.

Unit	Value
*T*, *K*	300
*ε_r_*	4
*c*_0_, cm^−3^	1.37 × 10^19^
*σ_max_*, S/m	9892
*d*, nm	506
*L*, μm	69.65
*W*, µm	57.65
